# PD-L1 Expression in Human Breast Cancer Stem Cells Is Epigenetically Regulated through Posttranslational Histone Modifications

**DOI:** 10.1155/2019/3958908

**Published:** 2019-02-21

**Authors:** Pramod Darvin, Varun Sasidharan Nair, Eyad Elkord

**Affiliations:** ^1^Cancer Research Center, Qatar Biomedical Research Institute, Hamad Bin Khalifa University, Qatar Foundation, Doha, Qatar; ^2^Institute of Cancer Sciences, University of Manchester, Manchester, UK

## Abstract

Tumor progression through immune evasion is a major challenge in cancer therapy. Recent studies revealed that enhanced PD-L1 expression in cancer stem cells is linked to immune evasion. Understanding the mechanisms behind this PD-L1 overexpression in cancer stem cells is critical for developing more effective strategies for preventing immune evasion and increasing the efficacy of anti-PD-1/PD-L1 therapy. Tumorsphere formation in breast cancer cells enhanced epithelial to mesenchymal transition (EMT), which is evident by increased expression of mesenchymal markers. In this study, we analyzed CpG methylation of PD-L1 promoter in MCF-7 and BT-549 breast cancer cells and tumorspheres derived from them. PD-L1 promoter was significantly hypomethylated in MCF-7 tumorspheres, but not from BT-549 tumorspheres, compared with their cell line counterparts. The active demethylation of PD-L1 promoter was confirmed by the increase in the distribution of 5hmC and decrease in 5mC levels and the upregulation of TET3 and downregulation of DNMTs enzymes in MCF-7 tumorspheres, compared with the cell line. Additionally, we checked the distribution of repressive histones H3K9me3, H3K27me3, and active histone H3K4me3 in the PD-L1 promoter. We found that distribution of repressive histones to the PD-L1 promoter was lower in tumorspheres, compared with cell lines. Moreover, an overexpression of histone acetylation enzymes was observed in tumorspheres suggesting the active involvement of histone modifications in EMT-induced PD-L1 expression. In summary, EMT-associated overexpression of PD-L1 was partially independent of promoter CpG methylation and more likely to be dependent on posttranslational histone modifications.

## 1. Introduction

Breast cancer is the most common cancer in women accounting for 30% of all new cases reported, and it is a major cause of cancer-related death [1]. Recent advances in early detection and therapeutic interventions reduced the mortality rate remarkably [1]. Cancer immunotherapy has recently shown promising results for treating different cancers. Immune checkpoint inhibitors, as immunotherapeutic agents, showed promising outcomes with higher overall survival rate and progression-free survival, but unfortunately this has been achieved in a small fraction of cancer patients [2]. Even though therapy resistance, recurrence, and metastasis are still major challenges in breast cancer therapy and management, it has been reported that the presence of a subset of cells with unique features like self-renewal and differentiation called cancer stem cells (CSCs) could be a major contributor towards these challenges [3].

Numerous studies reported the overexpression of programmed death-ligand 1 (PD-L1) as a predictive biomarker for differentiating responders and nonresponders undergoing immune checkpoint inhibition (ICI) therapies targeting programmed cell death-1 (PD-1)/PD-L1 [4–7]. Moreover, PD-L1 overexpression plays a critical role in immune evasion through increase of T-cell apoptosis in many cancers [8]. The overexpression of PD-L1 can also act as a molecular shield to protect tumor cells from T-cell mediated killing [9]. Additionally, PD-L1 overexpression in MC38 murine colon cancer cells showed a direct suppression of CD8^+^ TILs [10]. It has recently been reported that overexpression of PD-L1 in CSCs contributes to immune evasion through EMT/*β*-catenin/STT3/PD-L1 signaling axis [11]. The expression of PD-L1 is regulated through multiple signaling pathways and transcriptional control. The genetic modifications for constitutive overexpression of PD-L1 in tumors could be explained due to the copy number alterations and potential oncogenic signaling pathways [12, 13]. We have recently reported that PD-L1 is overexpressed in human tumor tissues and dual inhibition of signal transducers and activator of transcription 1 (STAT1) and STAT3 can downregulate PD-L1 expression [14–16]. However, epigenetic mechanisms behind the regulation of PD-L1 are not fully disclosed.

Understanding the regulatory mechanisms involved in PD-L1 expression may open possibilities for the development of combination strategies to improve the efficacy of PD-1/PD-L1 blockade therapies. DNA promoter methylation studies in breast and colon cancer patients using paired normal and tumor tissues showed hypomethylation irrespective of their PD-L1 expression status [15, 16]. It has been reported that treatment of breast cancer cells with demethylating agent azacytidine induced an upregulation in PD-L1 expression [17]. These data collectively indicate that apart from DNA methylation, multiple regulatory mechanisms might be involved in the expression of PD-L1.

In this study, we investigated epigenetic regulatory mechanisms involved in the PD-L1 expression associated with epithelial to mesenchymal transitions (EMT) in human breast cancer stem cells. We found that PD-L1 expression was significantly upregulated in MCF-7 and BT-549 tumorspheres, compared with cell lines, and this upregulation was partially dependent on the PD-L1 promoter demethylation. Moreover, our results showed that less distribution of repressive histones in the PD-L1 promoter region and overexpression of histone acetylation enzymes can also contribute to the PD-L1 upregulation in tumorspheres, compared with cell lines. These data shed light on the possible epigenetic mechanisms involved in the upregulation of PD-L1 in tumorspheres.

## 2. Materials and Methods

### 2.1. Cell and Tumorsphere Culture

MCF-7 and BT-549 breast cancer cell lines (ATCC, USA) were maintained in RPMI-1640 medium supplemented with 10% fetal bovine serum (FBS) and 1% penicillin/streptomycin (Sigma Aldrich, St. Louis, USA) in a humidified incubator at 37°C and 5% CO_2_. For BT-549 cells, the cells were maintained in the media supplemented with 0.023 IU/ml insulin (Sigma Aldrich).

For the generation of tumorspheres, 80-90% confluent cell plates were trypsinized, washed, and resuspended in Cancer Stem Premium™ medium (ProMab Biotechnologies, Richmond, CA, USA). 1x10^4^ cells/ml were cultured in Cancer Stem Premium™ media in ultra-low attachment Nunclon Sphera plate (Thermo Scientific, Nunclon Sphera, Roskilde, Denmark). The cells were incubated for 5 to 10 days for tumorsphere formation. Media change was done by collecting the tumorspheres in 15 ml falcon tubes and allowed to settle by gravity. The pellets were washed with 1X PBS. Tumorspheres older than seven days were used for the subsequent experiments.

### 2.2. Flowcytometry

MCF-7 and BT-549 cells were trypsinized and washed with 1X PBS. The tumorspheres formed from MCF-7 and BT-549 were collected and washed with 1X PBS following gravity sedimentation. Trypsinization was done for five to seven minutes with thorough mixing on every 30 seconds. After trypsinization, the single cell suspension was mixed with an equal amount of complete media and cells were collected by centrifuging at 1600 rpm for three minutes. The cells were resuspended in complete RPMI-1640 media and sieved using 40 *µ*m nylon cell strainers (STEMCELL technologies, Vancouver, Canada). 1x10^5^ cells were resuspended in 100 *µ*l staining buffer (phosphate-buffered saline (PBS) with 2% FCS and 0.1% sodium azide) in FACS tube and stained with PD-L1-APC (clone MIH1; eBioscience, San Diego, USA). Data were acquired on BD LSRFortessa flow cytometer using BD FACS Diva software (BD Biosciences, Oxford, UK) and analyzed on FlowJo version 10 software (Tree Star Inc., Oregon, USA).

### 2.3. Western Blotting

The expression levels of EMT markers were measured using western blotting. Briefly, MCF-7 and BT-549 cells and tumorspheres were lysed on ice using 1X RIPA lysis buffer (ThermoFisher Scientific, Massachusetts, USA) containing protease inhibitor cocktail (Sigma Aldrich) and phosphatase inhibitors. Protein concentrations were measured using the Pierce™ BCA Protein Assay kit (ThermoFisher Scientific) according to the manufacturer's instruction. The absorbance was measured using the FLUOstar Omega microplate reader at 660 nm (BMG LABTECH, Ortenberg, Germany). Equal amounts of proteins were resolved in 10% acrylamide gel and blotted on nitrocellulose membrane (Amersham Biosciences, Little Chalfont, UK). Membrane blocking was done by 5% skim milk or 5% Bovine serum albumin (BSA) in TBS-T. The membranes were then incubated overnight at 4°C with primary antibodies, *β*-actin, E-Cadherin, N-Cadherin, Vimentin, Snail, HDAC1, and HAT (p300/CBP). All primary antibodies were prepared at 1:1000 dilution in 5% skim milk or BSA in TBS-T. The membranes were then incubated with HRP-conjugated donkey anti-rabbit or Goat anti-mouse IgG (Invitrogen, California, USA) secondary antibodies (Dilution 1:10000) at room temperature for 2 h. Detection was performed using SuperSignal™ West Pico PLUS Chemiluminescent substrate (ThermoFisher Scientific, Massachusetts, USA) and images acquired using Molecular Imager® ChemiDoc™ XRS+ with Image Lab™ Software (Bio-Rad, California, USA). The densitometric analyses were performed using ImageJ software (National Institute of Health, Maryland, USA).

### 2.4. Quantitative Real-Time PCR

DNA and RNA were isolated using RNA/DNA/Protein purification plus kit (Norgen Bioteck Corp), as previously described [15]. 1*µ*g RNA from each sample was reverse transcribed into cDNA using QuantiTect Reverse Transcription Kit (Qiagen, Hilden, Germany). PCR reactions were performed using QuantStudio 7 Flex qPCR (Applied Biosystems, California, USA) using Fast SYBER Green Master Mix (Applied Biosystems). Data were analyzed as previously described [15]. The absolute expression of DNMTs and TETs in both cell lines and tumorspheres was checked by comparing the relative expression values of all genes normalized to relative expression normalized with *β*-actin. Primer sequences are provided in Table S1a.

### 2.5. CpG Methylation Analysis by Bisulfite Sequencing

The genomic DNA was extracted from tumorsphere and cell lines and treated with bisulfite using the EZ DNA Methylation Kit (Zymo Research, Irvine, CA, USA) as previously described [15]. The sequences of M13-reverse primer used for sequencing are provided in Table S1c.

### 2.6. Chromatin Immunoprecipitation (ChIP) Assay

Cells and tumorspheres were subjected to ChIP analysis using Magna ChIP A/G chromatin immunoprecipitation kit (Merck Millipore, MA, USA) according to the manufacturer's instructions. Briefly, nuclear extracts prepared and sonicated using Covaris S2 system (Covaris, MA, USA) to obtain DNA fragments ranging from 100 to 200 bp. The assay was performed as previously described [15]. Primer sequences are provided in Table S1d.

### 2.7. Methyl-DNA Immunoprecipitation (MeDIP) Assay

Genomic DNA was prepared from the MCF-7 and BT-549 cells and tumorspheres. The DNA was sonicated using Covaris S2 system (Covaris, MA, USA) to obtain DNA fragments ranging from 200 to 400 bp. The sheared DNA immunoprecipitated using the 5hmC and 5mC mAbs. Isotype-matched control Ab was used to check nonspecific bindings. The immune complexes were precipitated using Dynabeads (Invitrogen). Relative enrichment of PD-L1 promoter region in the precipitated DNA fragments was analyzed by qPCR. Primer sequences are provided in Table S1d.

### 2.8. Statistical Analyses

The data were analyzed using the Shapiro-Wilk normality test with paired t-test/Wilcoxon matched-pairs signed rank test using GraphPad Prism 6.0 (GraphPad Software Inc., California, USA). The significances were represented as *∗∗∗P*<0.001, *∗∗P*<0.01, *∗P*<0.05, and ns* P*>0.05. The data were represented as mean + standard error of the mean (SEM).

## 3. Results

### 3.1. The PD-L1 Expression Is Upregulated in Tumorspheres Enriched with Cancer Stem Cells

**Figure 1:**
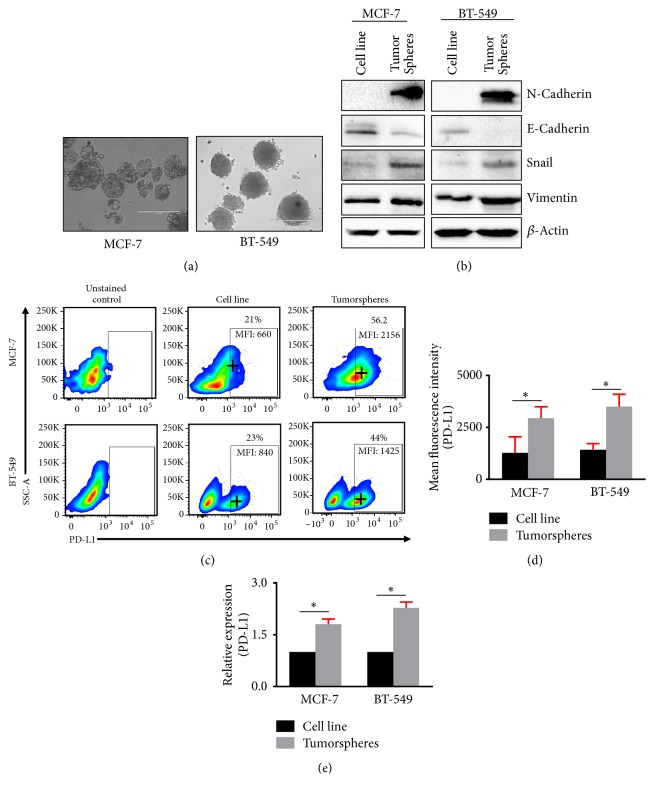
*EMT markers and PD-L1 expression in MCF-7 and BT-549 breast cancer cells and tumorspheres.* MCF-7 and BT-549 cells were cultured in Cancer Stem Premium™ media for 5-10 days. Representative image shows the tumorspheres formed from MCF-7 and BT-549 cell lines (a). Western blots show the expression of stemness markers in MCF-7 and BT-549 cell lines and tumorspheres (b). Representative flow cytometric plots show the expression of PD-L1 in MCF-7 and BT-549 cell lines and tumorspheres (c). Bar plots show the PD-L1 mean fluorescence intensity in MCF-7 and BT-549 cell lines and tumorspheres (d). Bar plots showing the relative expression of PD-L1 in MCF-7 and BT-549 cell lines and tumorspheres (e). All data were normalized to *β*-actin.

It has been reported that tumorsphere culture system provides a useful method for maintaining a CSC microenvironment [18]. In this study, we enriched the cells having stem cell property in MCF-7 and BT-549 cell lines using tumorsphere formation assay. Both MCF-7 and BT-549 cells showed typical tumorsphere formations (Figure 1(a)). We measured differential expression of the epithelial marker, E-Cadherin, and mesenchymal markers including vimentin, N-Cadherin, and snail to check the cancer stem cell properties of tumorspheres. Interestingly, we found that the expression of mesenchymal markers was upregulated, and epithelial marker E-Cadherin was downregulated in both MCF-7 and BT-549 tumorspheres, compared with cell lines (Figure 1(b)). The overexpression of mesenchymal markers shows epithelial to mesenchymal transition (EMT) happening in the tumorspheres. Moreover, we measured the mean fluorescence intensity (MFI) of immune checkpoint ligand, PD-L1, and found that PD-L1 was significantly upregulated in both tumorspheres, compared with cell lines (Figures 1(c) and 1(d)). mRNA analysis on cell lines and tumorspheres confirmed significant overexpression of PD-L1 in tumorspheres, compared with their cell line counterparts (Figure 1(e)). These data suggest that mesenchymal cells overexpress PD-L1, which might play an important role in immune evasion.

### 3.2. Tumorspheres Showed Distinct DNA Methylation Pattern for PD-L1 Promoter

**Figure 2:**
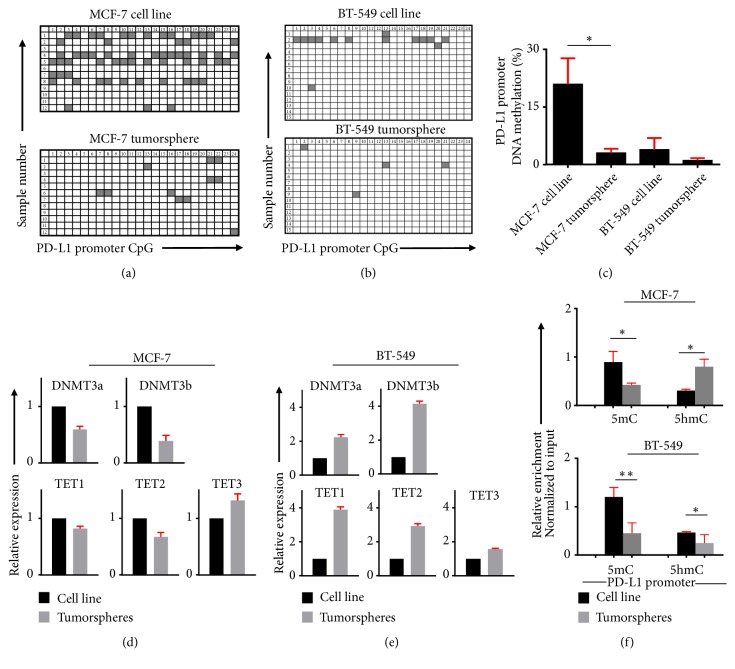
*Analysis of CpG methylation status and expression of methylation/demethylation enzymes in MCF-7 and BT-549 cell lines and tumorspheres*. Representative plots of PD-L1 promoter CpG methylation status analyzed by bisulfite sequencing of the genomic DNA isolated from MCF-7 (a) and BT-549 (b) cell lines and tumorspheres. Methylation status of individual CpG motif is shown by white (demethylation) or gray (methylation) colors. Bar plots show the methylation percentage of PD-L1 (c). Bar plots show the relative expression of DNMT3a, DNMT3b, TET1, TET2, and TET3 in MCF-7 (d) and BT-549 (e) cell lines and tumorspheres. All data were normalized to *β*-actin. Bar plots show the relative enrichment of 5-mC and 5-hmC in the PD-L1 promoter of MCF-7 (upper) and BT-549 (lower) cell lines and tumorspheres (f). Data are representative of two independent experiments.

The epigenetic changes involved in the PD-L1 expression during the EMT process were examined through PD-L1 promoter CpG methylation. CpG methylation plays a pivotal role in cancer initiation and progression [19]. This report prompted us to investigate the impact of DNA methylation on PD-L1 overexpression observed in tumorspheres. We analyzed 24 CpGs from the PD-L1 promoter to detect the CpG methylation landscape. Interestingly, we found that the PD-L1 promoter DNA methylation profile is different between MCF-7 and BT-549 tumorspheres. Tumorspheres from MCF-7 showed significant hypomethylation, compared with their cell line counterpart (Figures 2(a) and 2(c)), but there was no significant difference observed in between BT-549 cell line and tumorspheres (Figures 2(b) and 2(c)). PD-L1 promoter region was completely demethylated in both BT-549 cell line and tumorsphere (Figure 2(b)). These data suggest that overexpression of PD-L1 in tumorspheres is partially dependent on DNA methylation.

### 3.3. DNMTs Are Downregulated and TET3 Is Upregulated in MCF-7 Tumorspheres

The* de novo* DNMTs, DNMT3a, and DNMT3b are involved in the establishment of DNA methylation, whereas the TET proteins oxidize 5mC to generate 5hmC through active demethylation involving DNA repair machinery [20]. The balance between DNMTs and TETs can influence the gene expression through directly regulating the DNA methylation status [21]. The methylation/demethylation cycle was assessed in the breast cancer cells and tumorspheres through mRNA expression of DNMT3a, 3b, and TET1,2,3. Interestingly we found that, out of all three TETs, TET3 was increased in tumorspheres derived from both cell lines. The MCF-7 derived tumorspheres showed a decrease in DNMT3a and 3b suggests the involvement of DNA methylation-dependent epigenetic regulatory mechanism. Additionally, the increased levels of TET3 showed that a TET3 dependent active demethylation is active in MCF-7 tumorspheres (Figure 2(d)). The tumorspheres from BT-549 showed that both TETs and DNMTs were upregulated compared with the cell line. These data suggest that all cells were not following similar expression level of methylation/demethylation enzymes and promoter demethylation status for the upregulation of PD-L1 (Figure 2(e)). Moreover, the results were confirmed by evaluating 5hmC and 5mC levels in both cell lines and tumorspheres and found that MCF-7 derived tumorspheres enriched with cancer stem cells showed an increased 5hmC and decreased 5mC level, compared with cell line (Figure 2(f)), whereas tumorspheres from BT-549 show a significant decrease in both 5hmC and 5mC level, compared with the cell line. These data strongly recommend that active demethylation machinery is active in MCF-7 tumorspheres for the upregulation of PD-L1 expression but not in BT-549 tumorspheres.

### 3.4. Repressive Histones Regulate the Expression of PD-L1 in Tumorspheres

**Figure 3:**
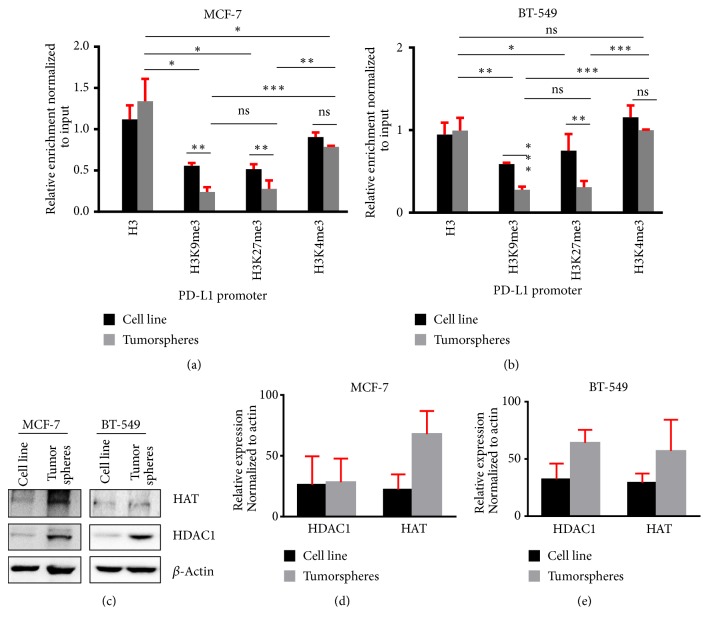
*Analysis of H3K9me3, H3K27me3, and H3K4me3 distribution in the PD-L1 promoter of MCF-7 and BT-549 cell lines and tumorspheres*. Chromatin prepared from MCF-7 and BT-549 cell lines and tumorspheres were precipitated using H3K9me3, H3K27me3, and H3K4me3 antibodies and IgG as negative control. qPCR was performed on the precipitated DNA using the PD-L1 primer and data were normalized to input. Bar plots show the H3K9me3, H3K27me3, and H3K4me3 distribution in MCF-7 (a) and BT-549 (b) PD-L1 promoters. Representative Western blots show the expression of HAT and HDAC in MCF-7 and BT-549 cell lines and tumorspheres (c). Bar plots show the HDAC1 and HAT in MCF-7 (d) and BT-549 (e) cell lines and tumorspheres. Data are representative of two independent experiments.

The epigenetic regulation of gene expression is not restricted to CpG hypomethylation but also depends on posttranslational modifications of histones. Histone modifications like methylation and acetylation are another epigenetic mechanism, which can regulate the chromatin organization [22]. To detect the role of histones in the regulation of PD-L1 expression, we checked the binding intensity of both repressive and active histone marks, including H3K9me3, H3K27me3, and H3K4me3 by keeping H3 as a positive control in the promoter region of PD-L1 in both breast cancer cells and tumorspheres. Despite the discrepancies in PD-L1 promoter CpG methylation and 5hmC pattern in tumorsphere-forming cancer stem cells, the histone modification showed a similar pattern in both cell lines. In both MCF-7 and BT-549 tumorspheres, the repressive histones H3K9me3 and H3K27me3 were significantly bound weakly to PD-L1 promoter, compared with their cell line counterparts (Figures 3(a) and 3(b)), whereas compared with repressive histones, positive regulatory histone H3K4me3 significantly binds more intensively to PD-L1 promoter in both tumorspheres and cell lines (Figure 3). These data suggest that, in tumorsphere-forming cells, the increase in PD-L1 expression is typically modulated through H3K9me3 and H3K27me3. Next, we checked histone acetylation machinery, as it is also an important regulator of chromatin anatomy. It has been reported that there is a dysregulation of HATs and HDACs involved in tumorigenesis [23]. HDAC1 is reported to be active under hypoxic conditions and in stem cells supporting their self-renewal [24]. We also observed a consistent overexpression of HDAC1 in tumorspheres derived from MCF-7 and BT-549 cells (Figures 3(c) and 3(d)). HAT (p300/CBP) is a transcriptional coactivator of histone acetyltransferase enzyme family that are responsible for epigenetic activation of EMT transcription factors, promoting breast cancer aggressiveness [25]. HAT was constitutively increased in the tumorspheres, compared with the cell line counterparts (Figures 3(c) and 3(e)). Altogether, our data suggest that both active histone acetylation and methylation play roles in the upregulation of PD-L1 in breast cancer stem cells.

## 4. Discussion

Cancer stem cells are the rare population of cells present in most of tumors, and these cells play critical roles in drug resistance, metastasis, recurrence, and immune evasion [26]. It has been reported that epigenetic silencing of antigen peptide transporter 1 (TAP1) gene in breast cancer stem cells promotes immune evasion [27]. Recent studies reported that expression of PD-L1 in the cancer cells is one of the major regulating factors for immune evasion [10, 28]. Also, EMT mediates immune evasion through the upregulation of multiple transcription factors and effector proteins. Moreover, upregulation of PD-L1 expression in CSCs made them resistant to peripheral blood mononuclear cells-mediated cancer cell killing* in vitro *[11]. Detailed mechanistic knowledge about the regulation of PD-L1 expression should help to avoid immune evasion as well as immunotherapy resistance. With this goal, we enriched the cancer stem cells through tumorsphere formation and the epigenetic regulatory mechanisms involved in the PD-L1 expression were investigated. We selected MCF-7 (luminal A subtype) and BT-549 (triple negative breast cancer, TNBC), which are known to maintain high degree of genetic mutations and epigenetic regulatory mechanisms [29]. However, role of tumor microenvironment in these epigenetic regulatory mechanisms and the expression of multiple proteins are the limiting factor of using cell lines.

PD-L1 expressions in cancer cells are regulated through multiple signaling cascades and mechanisms. We have recently reported that dual inhibitions of STAT1 and STAT3 constitutively inhibit PD-L1 expression in human breast cancer cells [14]. In addition to the Jak/STAT pathway, multiple other signaling cascades such as RAS/RAF/MEK/MAPK-ERK [30, 31], PI3K/PTEN/Akt/mTOR [32], EML4-ALK [33, 34], and EGFR signaling pathways [35–37] were shown to have regulatory effects on PD-L1 expression in multiple malignancies [38].

Recently, we have reported that the overexpression of PD-L1 in breast and colon cancer tissues is independent of promoter CpG methylation and repressor histone tri-methylation [15, 16]. The promoter CpG methylation analysis of TNBC cell line BT-549 and the CSC enriched tumorsphere revealed similar methylation profile. This result suggests that there could be an additional epigenetic/transcription factor-mediated regulation for PD-L1 expression. The involvement of multiple regulatory mechanisms in the expression of PD-L1 during the EMT is also reported in nonsmall cell lung carcinoma [39]. In this study, we showed that the CpG methylation patterns in MCF-7 cell line and tumorsphere were different from BT-549. In MCF-7, a significant difference in DNA methylation pattern was detected with more hypomethylation in tumorspheres than the cell line. In addition to the CpG methylation, the expressions of DNMTs and TETs were also different between the two cell lines. Similar results were observed in the 5hmC and 5mC distribution. These indicate that cancer stem cells have different epigenetic regulatory mechanisms depending on the physiological and molecular status of cancer.

The posttranslational methylation of histones at the N-terminal tail has high importance in the protein expression. Histone 3 lysine 9 and 27 tri-methylation (H3K9me3, H3K27me3) leads to the inhibition of gene expressions [40]. ChIP-qPCR analysis on the PD-L1 promoter in both MCF-7 and BT-549 cell lines showed significantly decreased levels of H3K9me3 and H3K27me3 in tumorspheres, compared with the cell lines (Figures 3(a) and 3(b)). Interestingly, the H3K4me3 did not show significant change between cell lines and tumorspheres. This indicates that the upregulation of PD-L1 expression in cancer stem cells could be controlled through histone modifications. In addition to histone methylation, histone acetylation through HAT and HDAC has a major role in the gene expression through modulation of chromatin structure and enabling transcription factor binding, leading to the increased gene expression.

## 5. Conclusions

**Figure 4:**
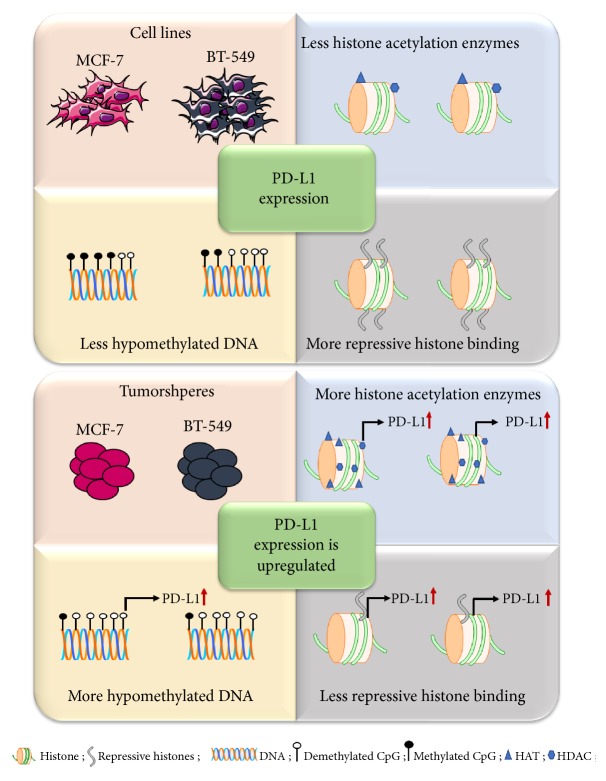
*A schematic diagram summarizing the epigenetic events involved in the regulation of PD-L1 expression in MCF-7 and BT-549 cell lines and tumorspheres*. The PD-L1 promoter CpG is hypomethylated in tumorspheres. Moreover, distribution of repressive histones (H3K27me3 and H3K9me3) in the PD-L1 promoter is decreased along with an upregulation of histone acetylation enzymes in tumorspheres, compared with cell lines.

In this study, we report that epigenetic modifications including DNA methylation and posttranslational histone modifications (methylation and acetylation) regulated the expression of PD-L1 in breast cancer stem cells. Alterations in expression of methylation and demethylation enzymes were detected in the cell lines and tumorspheres. Moreover, histone modifications such as lysine tri-methylation and acetylation play significant roles in the upregulation of PD-L1 expression in CSC. The overall conclusion is graphically represented in Figure 4. Further studies are needed to validate the impact of DNA copy number variations in epigenetic regulations.

## Data Availability

No data were used to support this study.

## Supplementary Materials

Supplementary Materials Table S1: Primer sequences used in this study.

## References

[1] Siegel R. L., Miller K. D., Jemal A. (2018). Cancer statistics, 2018. *CA: A Cancer Journal for Clinicians*.

[2] Thallinger C., Füreder T., Preusser M. (2018). Review of cancer treatment with immune checkpoint inhibitors: current concepts, expectations, limitations and pitfalls. *Wiener Klinische Wochenschrift*.

[3] Tan B. T., Park C. Y., Ailles L. E., Weissman I. L. (2006). The cancer stem cell hypothesis: a work in progress. *Laboratory Investigation*.

[4] Patel S. P., Kurzrock R. (2015). PD-L1 expression as a predictive biomarker in cancer immunotherapy. *Molecular Cancer Therapeutics*.

[5] Mathew M., Safyan R. A., Shu C. A. (2017). PD-L1 as a biomarker in NSCLC: challenges and future directions. *Annals of Translational Medicine*.

[6] Aguiar P. N., De Mello R. A., Hall P., Tadokoro H., De Lima Lopes G. (2017). PD-L1 expression as a predictive biomarker in advanced non-small-cell lung cancer: updated survival data. *Immunotherapy*.

[7] Teng F., Meng X., Kong L., Yu J. (2018). Progress and challenges of predictive biomarkers of anti PD-1/PD-L1 immunotherapy: a systematic review. *Cancer Letters*.

[8] Dong H., Strome S. E., Salomao D. R. (2002). Tumor-associated B7-H1 promotes T-cell apoptosis: a potential mechanism of immune evasion. *Nature Medicine*.

[9] Zou W., Wolchok J. D., Chen L. (2016). PD-L1 (B7-H1) and PD-1 pathway blockade for cancer therapy: mechanisms, response biomarkers, and combinations. *Science Translational Medicine*.

[10] Juneja V. R., McGuire K. A., Manguso R. T. (2017). PD-L1 on tumor cells is sufficient for immune evasion in immunogenic tumors and inhibits CD8 T cell cytotoxicity. *The Journal of Experimental Medicine*.

[11] Hsu J., Xia W., Hsu Y. (2018). STT3-dependent PD-L1 accumulation on cancer stem cells promotes immune evasion. *Nature Communications*.

[12] Inoue Y., Yoshimura K., Mori K. (2016). Clinical significance of *PD-L1* and *PD-L2* copy number gains in non-small-cell lung cancer. *Oncotarget*.

[13] Lastwika K. J., Wilson W., Li Q. K. (2016). Control of PD-L1 expression by oncogenic activation of the AKT-mTOR pathway in non-small cell lung cancer. *Cancer Research*.

[14] Sasidharan Nair V., Toor S. M., Ali B. R., Elkord E. (2018). Dual inhibition of STAT1 and STAT3 activation downregulates expression of PD-L1 in human breast cancer cells. *Expert Opinion on Therapeutic Targets*.

[15] Sasidharan Nair V., El Salhat H., Taha R. Z., John A., Ali B. R., Elkord E. (2018). DNA methylation and repressive H3K9 and H3K27 trimethylation in the promoter regions of PD-1, CTLA-4, TIM-3, LAG-3, TIGIT, and PD-L1 genes in human primary breast cancer. *Clinical Epigenetics*.

[16] Sasidharan Nair V., Toor S. M., Taha R. Z., Shaath H., Elkord E. (2018). DNA methylation and repressive histones in the promoters of PD-1, CTLA-4, TIM-3, LAG-3, TIGIT, PD-L1, and galectin-9 genes in human colorectal cancer. *Clinical Epigenetics*.

[17] Li H., Chiappinelli K. B., Guzzetta A. A. (2014). Immune regulation by low doses of the DNA methyltransferase inhibitor 5-azacitidine in common human epithelial cancers. *Oncotarget*.

[18] Zhu Z., Chen L., Liu J. (2017). A novel three-dimensional tumorsphere culture system for the efficient and low-cost enrichment of cancer stem cells with natural polymers. *Experimental and Therapeutic Medicine*.

[19] Kulis M., Esteller M. (2010). DNA methylation and cancer. *Advances in Genetics*.

[20] Pastor W. A., Aravind L., Rao A. (2013). TETonic shift: biological roles of TET proteins in DNA demethylation and transcription. *Nature Reviews Molecular Cell Biology*.

[21] Nair V. S., Song M. H., Ko M., Oh K. I. (2016). DNA demethylation of the Foxp3 enhancer is maintained through modulation of ten-eleven-translocation and DNA methyltransferases. *Molecules and Cells*.

[22] Handy D. E., Castro R., Loscalzo J. (2011). Epigenetic modifications: basic mechanisms and role in cardiovascular disease. *Circulation*.

[23] Liu N., Li S., Wu N., Cho K. (2017). Acetylation and deacetylation in cancer stem-like cells. *Oncotarget*.

[24] Jamaladdin S., Kelly R. D. W., O'Regan L. (2014). Histone deacetylase (HDAC) 1 and 2 are essential for accurate cell division and the pluripotency of embryonic stem cells. *Proceedings of the National Acadamy of Sciences of the United States of America*.

[25] Cho M., Park J., Choi H. (2015). DOT1L cooperates with the c-Myc-p300 complex to epigenetically derepress CDH1 transcription factors in breast cancer progression. *Nature Communications*.

[26] Batlle E., Clevers H. (2017). Cancer stem cells revisited. *Nature Medicine*.

[27] Sultan M., Vidovic D., Paine A. S. (2018). Epigenetic silencing of TAP1 in aldefluor(+) breast cancer stem cells contributes to their enhanced immune evasion. *Stem Cells*.

[28] Wang Y., Wang H., Yao H., Li C., Fang J., Xu J. (2018). Regulation of PD-L1: emerging routes for targeting tumor immune evasion. *Frontiers in Pharmacology*.

[29] Gillet J., Varma S., Gottesman M. M. (2013). The clinical relevance of cancer cell lines. *JNCI Journal of the National Cancer Institute*.

[30] Loi S., Dushyanthen S., Beavis P. A. (2016). RAS/MAPK activation is associated with reduced tumor-infiltrating lymphocytes in triple-negative breast cancer: therapeutic cooperation between MEK and PD-1/PD-L1 immune checkpoint inhibitors. *Clinical Cancer Research*.

[31] Sumimoto H., Takano A., Teramoto K., Daigo Y., Chellappan S. (2016). RAS–mitogen-activated protein kinase signal is required for enhanced PD-L1 expression in human lung cancers. *PLoS ONE*.

[32] Parsa A. T., Waldron J. S., Panner A. (2007). Loss of tumor suppressor PTEN function increases B7-H1 expression and immunoresistance in glioma. *Nature Medicine*.

[33] Koh J., Jang J., Keam B. (2015). EML4-ALK enhances programmed cell death-ligand 1 expression in pulmonary adenocarcinoma via hypoxia-inducible factor (HIF)-1*α* and STAT3. *OncoImmunology*.

[34] Ota K., Azuma K., Kawahara A. (2015). Induction of PD-L1 expression by the EML4-ALK oncoprotein and down stream signaling pathways in non-small cell lung cancer. *Clinical Cancer Research*.

[35] Azuma K., Ota K., Kawahara A. (2014). Association of PD-L1 overexpression with activating EGFR mutations in surgically resected nonsmall-cell lung cancer. *Annals of Oncology*.

[36] Akbay E. A., Koyama S., Carretero J. (2013). Activation of the PD-1 pathway contributes to immune escape in EGFR-driven lung tumors. *Cancer Discovery*.

[37] Rech A. J., Vonderheide R. H. (2013). Dynamic interplay of oncogenes and T cells induces PD-L1 in the tumor microenvironment. *Cancer Discovery*.

[38] Zerdes I., Matikas A., Bergh J., Rassidakis G. Z., Foukakis T. (2018). Genetic, transcriptional and post-translational regulation of the programmed death protein ligand 1 in cancer: biology and clinical correlations. *Oncogene*.

[39] Asgarova A., Asgarov K., Godet Y. (2018). PD-L1 expression is regulated by both DNA methylation and NF-kB during EMT signaling in non-small cell lung carcinoma. *OncoImmunology*.

[40] Leszinski G., Gezer U., Siegele B., Stoetzer O., Holdenrieder S. (2012). Relevance of histone marks H3K9me3 and H4K20me3 in cancer. *Anticancer Reseach*.

