# Supplementary material for: PD-L1 Expression in Human Breast Cancer Stem Cells Is Epigenetically Regulated through Posttranslational Histone Modifications
Source: J Oncol. 2019 Feb 21;2019:3958908. doi: 10.1155/2019/3958908 (PMC6409026; doi:10.1155/2019/3958908)
Supplement: Supplementary Materials — Table S1: Primer sequences used in this study. [file 3958908.f1.pdf]

Table S1: Primer sequences used in this study

a) RT-qPCR primers

Genes	Forward primer (5′-3′)	Reverse primer (5′-3′)
PD-L1	TGGCATTTGCTGAACGCATTT	TGCAGCCAGGTCTAATTGTTTT
TET-1	CATCAGTCAAGACTTTAAGCCCT	CGGGTGGTTTAGGTTCTGTTT
TET-2	GATAGAACCAACCATGTTGAGGG	TGGAGCTTTGTAGCCAGAGGT
TET-3	GCCGGTCAATGGTGCTAGAG	CGGTTGAAGGTTTCATAGAGCC
DNMT3a	CCGATGCTGGGGACAAGAAT	CCCGTCATCCACCAAGACAC
DNMT3b	AGGGAAGACTCGATCCTCGTC	GTGTGTAGCTTAGCAGACTGG
β-ACTIN	AGAGCTACGAGCTGCCTGAC	AGCACTGTGTTGGCGTACAG

b) PCR primers for CpG methylation analysis

Genes	Forward primer (5′-3′)	Reverse primer (5′-3′)
PD-L1 promoter	TTGTTTTGGGTAGAGGTGGG	AAAAACCAAATACATTACCTATTCTTA

c) Sequencing Primer

Genes	Forward primer (5′-3′)	Reverse primer (5′-3′)
M-13	TGTAAAACGACGGCCAGT	CAGGAAACAGCTATGACC

d) ChIP-qPCR primers

Genes	Forward primer (5′-3′)	Reverse primer (5′-3′)
PD-L1 promoter	TATTTATAAGGTGGAAGTTTTG AGG	TGCCCAAGGCAGCAAATCCAG
